# Activation of Brainstem Pro-opiomelanocortin Neurons Produces Opioidergic Analgesia, Bradycardia and Bradypnoea

**DOI:** 10.1371/journal.pone.0153187

**Published:** 2016-04-14

**Authors:** Serena Cerritelli, Stefan Hirschberg, Rob Hill, Nina Balthasar, Anthony E. Pickering

**Affiliations:** 1 School of Physiology, Pharmacology & Neuroscience, Biomedical Sciences Building, University of Bristol, Bristol, BS8 1TD, United Kingdom; 2 Department of Anaesthesia, University Hospitals Bristol, Bristol, BS2 8HW, United Kingdom; University of California, Los Angeles, UNITED STATES

## Abstract

Opioids are widely used medicinally as analgesics and abused for hedonic effects, actions that are each complicated by substantial risks such as cardiorespiratory depression. These drugs mimic peptides such as β-endorphin, which has a key role in endogenous analgesia. The β-endorphin in the central nervous system originates from pro-opiomelanocortin (POMC) neurons in the arcuate nucleus and nucleus of the solitary tract (NTS). Relatively little is known about the NTS_POMC_ neurons but their position within the sensory nucleus of the vagus led us to test the hypothesis that they play a role in modulation of cardiorespiratory and nociceptive control. The NTS_POMC_ neurons were targeted using viral vectors in a POMC-Cre mouse line to express either opto-genetic (channelrhodopsin-2) or chemo-genetic (Pharmacologically Selective Actuator Modules). Opto-genetic activation of the NTS_POMC_ neurons in the working heart brainstem preparation (n = 21) evoked a reliable, titratable and time-locked respiratory inhibition (120% increase in inter-breath interval) with a bradycardia (125±26 beats per minute) and augmented respiratory sinus arrhythmia (58% increase). Chemo-genetic activation of NTS_POMC_ neurons *in vivo* was anti-nociceptive in the tail flick assay (latency increased by 126±65%, p<0.001; n = 8). All effects of NTS_POMC_ activation were blocked by systemic naloxone (opioid antagonist) but not by SHU9119 (melanocortin receptor antagonist). The NTS_POMC_ neurons were found to project to key brainstem structures involved in cardiorespiratory control (nucleus ambiguus and ventral respiratory group) and endogenous analgesia (periaqueductal gray and midline raphe). Thus the NTS_POMC_ neurons may be capable of tuning behaviour by an opioidergic modulation of nociceptive, respiratory and cardiac control.

## Introduction

Opioids have been used for several thousand years for their potent mood altering and analgesic properties [2]. These opioid drugs mimic the actions of endogenous peptide neurotransmitters including the endorphin, enkephalin and dynorphin families acting at mu, delta and kappa opioid receptors [3–5]. One of the best characterised of these peptides, β-endorphin [6], has been shown to play roles in endogenous analgesia and stress responses [7] as well as being linked to reward processing [8], feeding [9] and cardiorespiratory regulation [10]. The selective genetic ablation of β-endorphin produces a specific deficit in stress-induced analgesia [11].

Given their analgesic actions, considerable effort has been invested to delineate the characteristics of the opioid peptides and receptor systems with the aim of identifying new targets that may be free of limiting side effects such as cardiorespiratory depression [3]. In contrast with this extensive understanding of opioids and receptors there is considerably less knowledge of the organisation and functional behaviour of β-endorphin releasing neurons.

β-endorphin is a cleavage peptide product of the precursor pro-opiomelanocortin (POMC) which is also the precursor of a number of melanocortin peptides such as adrenocorticotrophic hormone and the melanocyte stimulating hormones (including α-MSH). These peptides are differentially produced within neurons by expression of specific cleavage enzymes [12]. POMC is synthesised in neurons located in two main clusters in the mammalian brain—in the arcuate nucleus of the hypothalamus (ARC) and a smaller group of neurons in the nucleus of the solitary tract (NTS) [13–17]. The POMC neurons of the ARC have been shown to play a key role in the regulation of feeding behaviour and energy balance [18]. These actions are mediated predominantly by α-MSH acting via melanocortin-4-receptors [19, 20]. In contrast, the role of the NTS cluster of POMC neurons in metabolic control is less well defined. Their long-term inhibition has little detectable effect on metabolic control or weight regulation, although they seem to play an acute role in satiety [21].

Intriguingly, an opioid-mediated analgesic action can be elicited by stimulation of the NTS or the vagus [10, 22, 23]. There is also evidence that activation of opioid receptors in the NTS can exert potent effects on cardiorespiratory control. This has led to the proposition that opioidergic systems may target the NTS to regulate both nociceptive and autonomic function [10, 24, 25], however, the chemo-architectural complexity and cellular heterogeneity of the NTS has impeded further exploration of this hypothesis. Therefore to test this hypothesis, we used selective opto- and chemo-genetic [1, 26] techniques in POMC-Cre mice [27] to test the hypothesis that the NTS_POMC_ neurons modulate cardiorespiratory and nociceptive processing, by an opioidergic action. We show that this small cluster of several hundred NTS_POMC_ neurons extend extensive projections to key autonomic and pain control regions within the brainstem to exert a potent influence on cardiorespiratory control and on nociceptive processing, which is mediated by opioid release.

## Materials and Methods

### Ethical Approval

All procedures conformed to the UK Animals (Scientific Procedures) Act of 1986 and were approved by the University of Bristol ethical review committee.

### Animals

Experiments were performed on adult male and female POMC-Cre-GFP mice. These mice were bred by crossing heterozygous POMC-Cre mice [27], to homozygous B6.Cg-*Gt(ROSA)26Sor*^*tm3(CAG-EYFP)Hze*^/J mice (The Jackson Laboratory, stock number 007903). Animals were housed using a standard 12 h light/dark cycle with *ad libitum* access to food and water.

### Stereotaxic Injection of Viral Vectors

Adult mice were anaesthetised using ketamine (70mg/kg, i.p) and medetomidine (0.5mg/kg, i.p) before being positioned in a stereotaxic frame with head angled down (20°). A midline skin incision was made over the dorsal surface of the skull to expose the atlanto-occipital membrane which was also incised. *Calamus scriptorius* on the dorsal surface of the medulla was used as a reference landmark for the stereotaxic injections. The vector injection procedure used similar methods to Howorth [28] and Hickey [29] and is recapitulated here in brief. The AAV vectors were drawn up into a mineral oil-filled pulled microcapillary pipette with a tip diameter of 20μm. The pipette was inserted at a 35° angle to the vertical axis aiming rostrally, positioned 200μm lateral to the midline at the rostrocaudal level of *calamus scriptorius*. Microinjections (180nl, over 2 minutes) were made at 4 sites along a single track at the following depths: 1.00, 0.75, 0.50 and 0.25mm below the surface of the medulla. The pipette was left in place for 2 min after each injection. Following viral vector injection the skin was sutured. Atipamezole (1mg/kg, i.p) was administered to reverse anaesthesia and buprenorphine (0.1mg/kg, s.c.) for analgesia. Animals made a rapid recovery after vector injection and any mouse failing to show normal locomotor activity, weight loss >15% or failure to self-care was humanely euthanized (pentobarbital sodium 100mg/kg, i.p). All experiments were performed 3–4 weeks after AAV vector delivery.

### Viral Vectors

Three vectors were used in the course of these experiments:

- AAV-EF1α-DIO-ChR2-mCherry (UNC Vector Core Services, Chapel Hill, NC; 6x10^12^ viral particles/ml) was microinjected unilaterally for opto-genetic working heart-brainstem preparation (WHBP) and anatomical tracing experiments. For control animals used in nociceptive testing, this vector was injected bilaterally.
- AAV-hSyn-FLEX-Synaptophysin-mCherry (from David Olson, University of Michigan Hospital and Health Systems, MI, USA) was microinjected bilaterally into the NTS. The same gene insert has previously been used in adenoviral vectors for tracing studies [30, 31]. This vector had a titre of 3x10^12^ virus particles/ml and was diluted 1:1 in sterile PBS on the day of the experiment.
- AAV-hSyn-FLEX-PSAM-5HT_3_ (from Scott Sternson, Janelia Farm Research Campus, VA, USA) was used for the chemo-genetic studies and was microinjected bilaterally into the NTS. The viral titre was 2.8x10^12^ genome copies/ml and stock was diluted 1:10 with sterile PBS.

### Working Heart-Brainstem Preparation

WHBP experiments were performed on AAV-EF1α-DIO-ChR2-mCherry-injected animals. Full details of the WHBP procedures have been previously described [32] and are repeated here in brief. Mice (25-35g) were deeply anaesthetised with halothane (5% in O_2_), then bisected sub-diaphragmatically and exsanguinated. The head and thorax were cooled by immersion in 5°C Ringer’s solution (composition (in mM): 125 NaCl, 24 NaHCO_3_, 3 KCl, 2.5 CaCl_2_, 1.25 MgSO_4_, 1.25 KH_2_PO_4_, and 10 dextrose, pH 7.35–7.4 after carbogenation). The preparation was decerebrated at the pre-collicular level. The phrenic nerves and descending aorta were dissected free of connective tissue and the lungs removed. The ribcage and spinal cord were cut at T8. The cerebellum was removed to expose the dorsal surface of the medulla.

After transfer to the recording chamber, the preparation was positioned prone and a double lumen cannula was inserted into the descending aorta for retrograde perfusion with carbogen-gassed (95% O_2_, 5% CO_2_) Ringer’s solution containing Ficoll-70 (1.25%) at 31°C. The second lumen was used to monitor the perfusion pressure in the aorta via a pressure transducer (custom built, Physiology & Pharmacology electronic workshop, University of Bristol, UK). Continuous flow of perfusate was generated from a recycling reservoir (200ml) by a peristaltic pump (Watson-Marlow 505U, Cornwall, UK), at a flow rate of ~11ml/min. The heart resumed beating, usually within 1 min after cannulation as the preparation warmed, and rhythmic respiratory muscle contractions were observed within 2–5 min. The preparation was paralysed by adding a muscle relaxant to the perfusate (vecuronium, 200μg; Norcuron, Cambridge, UK).

Phrenic nerve activity was recorded using a glass bipolar suction electrode to monitor central respiratory activity. The preparation was tuned to obtain a robust eupnoeic pattern of phrenic nerve activity by adjusting perfusate flow and vascular resistance (with addition of vasopressin (100-200nM)) to obtain a perfusion pressure of ~70mmHg. Once the preparation had been tuned, little further adjustment was usually required and preparations remained stable for recordings for a further 2–4 h. Preparations that were not stable, as determined by a loss of the peripheral chemoreflex or arterial baroreflex responses, were excluded from analysis.

At the end of each WHBP experiment, the brainstem was immersed in formaldehyde (4% in 0.1M PB) for 48 h before transfer to PBS-azide (0.02% azide in 0.1M PBS) until required for histological analysis (see below).

### Data acquisition

The ECG was recorded using three 23-gauge needle electrodes. Custom built AC amplifiers (Physiology & Pharmacology electronic workshop, University of Bristol, UK) were used to record phrenic nerve activity and ECG. The signals were AC amplified (phrenic, x10-20 k; ECG, x5k) and band pass filtered (phrenic, 500 Hz to 3kHz; ECG, 50Hz to 3kHz). Instantaneous heart rate was triggered from the R wave of the ECG signal using a window discriminator. Signals were collected using an analogue to digital converter (micro1401, CED, Cambridge, UK) connected to a computer running Spike2 software (CED); data was acquired and displayed using custom scripts in Spike2.

### Opto-stimulation of NTS_POMC_ neurons

Opto-stimulation of NTS ChR2-mCherry transduced neurons was achieved by positioning a 200μm optic fibre (FT200-UMT 0.39 numerical aperture, Thorlabs, Newton, NJ) vertically over the dorsal surface of the medulla positioned at 200μm lateral and 200μm rostral to *calamus scriptorius*, on the side of prior vector microinjection. The light-source was a 445nm LED laser (PhoxX 445, Omicron, Taunusstein, Germany), which was controlled by Omicron Control Centre software (v2.1.4). The transduced cells were driven with 20ms light pulses at 2-20Hz and 0.6–9.1mW (exiting the fibre). The optic fibre was calibrated before each experiment using an optical power meter (PM100D, Thorlabs, Dachau, Germany).

To allow aggregation of the cardiovascular and respiratory responses, a ‘standard stimulation’ was used: the optic fibre was driven with light pulses (20ms) at 10Hz with an intensity of 4.6mW for 10s. These parameters were chosen as the standard stimulation because it repeatedly evoked cardiorespiratory responses in pilot experiments in the WHBP. The effects of opto-stimulation were assayed on heart rate, phrenic nerve activity and perfusion pressure and the response was considered stable when three repeated stimuli produced reproducible cardiorespiratory responses. To investigate the dose-dependency of responses, the NTS was illuminated with pulses (20ms) at a fixed intensity of 4.6mW, with varying frequencies (2Hz, 5Hz, 10Hz and 20Hz) or at a fixed frequency of 10Hz, with graded intensities relative to the standard stimulus (12.5%, 25%, 50% and 200%). A sham stimulus was also used to act as a control period (no illumination). To prevent desensitisation of ChR2 (Lin, 2011), all stimulations were separated by a minimum of 2–3 min.

### Cardiorespiratory analysis

Grouped data are presented as heart rate (bpm), interbreath interval (s) and respiratory sinus arrhythmia (RSA, bpm). The heart rate response was analysed by comparing the mean heart rate of a 10s baseline period, immediately before the stimulation commenced, and the minimum heart rate value during the stimulation period. The respiratory response was analysed by comparing the longest interbreath interval during the response (the apnoea) with the baseline interbreath interval (the mean of nine interbreath intervals immediately before the stimulation began). RSA was measured by comparing the mean baseline RSA before with the mean RSA during and into the period immediately following the stimulation. Each mean RSA was calculated by taking the peak-to-trough variations in heart rate across 4 consecutive respiratory cycles. In all cases this was also referenced against a period of sham stimulation (no illumination).

### Nociceptive Testing

Nociceptive testing was performed using the tail-flick test on POMC-Cre-GFP mice that had received NTS microinjections of either AAV-hSyn-FLEX-PSAM-5HT_3_ (PSAM mice) or AAV-EF1α-DIO-ChR2-mCherry (control vector; ChR2-control mice). Following surgery, the experimenter was blinded to the group allocation.

For the tail-flick test, mice were restrained by scruffing and a 2cm distal portion of the tail was immersed into a 52°C temperature-controlled water bath (custom built by the Physiology & Pharmacology electronic workshop, University of Bristol, UK). Mice responded with an abrupt withdrawal movement of the tail and the ‘tail-flick latency’ was recorded. The assay was repeated at 15 min intervals to prevent sensitisation and a 20s cut-off point to end the assay was chosen to prevent tissue damage.

An i.p. injection of the PSEM^89S^ ligand, was used to activate PSAM-5HT_3_ receptors [1]. PSEM^89S^ was dissolved in sterile saline (at 9.2mM or 27.5mM) on the day of the experiment. The experimenter was blinded to the identity of the drug/vehicle. Three baseline latencies were measured before i.p. injection of PSEM^89S^ (30mg/kg or 90mg/kg) or saline. Four measurements were then taken over the following hour (every 15 min) to measure the time-course of drug action. In some experiments, naloxone was injected (1mg/kg i.p.) 10 min prior to PSEM^89S^ injection. Naloxone hydrochloride (Tocris, Bristol, UK) was made up on the day of the experiment in sterile saline to a concentration of 550μM.

Data are presented as a percentage change in tail-flick latency from baseline, which was calculated as the mean tail-flick latency of the first and second tail-flick latencies before drug dosing. The third baseline measurement was included in graphs to display a time-course of the effect of PSEM^89S^.

### Tissue fixation

All mice for histological analysis were killed with a lethal dose of pentobarbital sodium (Euthatal, 100mg/kg, i.p.) and transcardially perfused with 18ml of formaldehyde (4% in 0.1M PB, pH 7.4). The brain was removed and post-fixed overnight and then transferred to a cryoprotectant (30% sucrose in 0.1M PB) for a minimum of 24h at 4°C.

### Immunohistochemistry

Brains were coronally sectioned on a freezing microtome (at 40μm intervals) into 4 series and free-floating immunohistochemistry was performed at room temperature on a shaking platform (RA Lamb, UK). Sections were washed 3 times in 0.1M PB for 10 min and permeabilised in ethanol (50% in distilled H_2_O) for 30 min. 3 additional washes were then done in 0.1M PB for 10 min. The tissue was incubated overnight with primary antibodies against GFP (polyclonal chicken anti-GFP, 1:5000; Abcam, UK), mCherry (monoclonal rat anti-RFP, 1:4000; Chromotek, Planegg, Germany) or ChAT (polyclonal goat anti-ChAT, 1:400; Merck Millipore, Nottingham, UK) in 0.1M PB containing 5% horse serum (Sigma, UK) and 0.3% Triton X-100 (Sigma, Dorset, UK). Sections were washed 3 times, then incubated for 3-4h with appropriate secondary antibodies conjugated to fluorophores (Alexa Fluor 488 and Alexa Fluor 594, 1:1000; Invitrogen, Warrington, UK) in 0.1M PB containing 2% horse serum and 0.3% Triton X-100. For experiments requiring dual immunohistochemistry for mCherry and ChAT, an anti-rat biotinylated secondary antibody (1:500; Vector Laboratories, Peterborough, UK) was used followed by a 3h tertiary incubation of Streptavidin Alexa Fluor 594 (1:1000; Invitrogen, Warrington, UK). Sections were washed and mounted onto electrostatically charged glass microscope slides (Superfrost Plus slides, Menzel-Glaser; Fisher Scientific, Loughborough, UK) and cover slipped (Menzel-Glaser; Fisher Scientific, Loughborough, UK) with fluorescence mounting media (FluorSave Reagent; Merck Millipore, Nottingham, UK). Negative controls were routinely run by omitting primary antibodies.

### Microscopy

Sections were examined using a Zeiss Axioskop 2 fluorescence microscope (Zeiss, Oberkochen, Germany) with a pE-2 LED excitation system (CoolLED, UK) light source. The excitation LEDs and excitation-emission filter cubes used were: (i) excitation LED 490nm/filter cube #10 (Carl Zeiss) was used for Alexa Fluor 488; (ii) excitation LED 565nm/custom made filter cube (excitation 560/40nm, dichroic 585nm, emission 630/75nm (Zeiss)) was used for Alexa Fluor 594/mCherry. Images were captured using an AxioCam camera and processed using Axiovision 4.7 software (Carl Zeiss, Cambridge, UK). Brightfield/darkfield images of sections were also captured to identify their rostro-caudal position.

Confocal microscopy and imaging was performed on selected areas of the medulla to obtain z-stacks, which allowed better visualisation of potential co-localisation of ChAT immuno-reactivity with mCherry. A Leica SP5-AOBS confocal laser scanning microscope (Leica Microsystems, Milton Keynes, UK) was used to take confocal stacks. Green fluorescent labelling (Alexa Fluor 488) was visualised with a 100mW Argon laser (488nm). Red fluorescent labelling (native mCherry or Alexa Fluor 594) was visualised with a 2mW Orange HeNe 594nm laser. Images were taken using x20 and x40 oil immersion objectives and Leica LAS AF software. A line average of 4 was used to reduce background noise.

### Figure preparation

For figures, phrenic nerve activity was rectified and smoothed (time constant = 50ms). Microscope images were optimised for brightness/contrast where necessary using Fiji ImageJ [33]. To present a large area of a coronal section of the medulla at a suitable magnification to see neuronal fibres, several images were taken at the same exposure and stitched together using Fiji ImageJ. All figures were prepared and annotated in Adobe Illustrator CS5 software.

### Histological Analysis

The distribution of Syp-mCherry puncta, revealed after mCherry immunohistochemistry, was mapped throughout the brainstem. A total of 8 coronal sections across the medulla of each brain were visualised (bregma -6.48mm to -8.12mm) and the density was mapped onto the corresponding section from the mouse brain atlas (Franklin & Paxinos, 2007). The densities of puncta in each region were rated on a numerical scale as 0—absent, 1—moderate, 2—high or 3—very high. The density in each region was averaged for each of the 8 rostro-caudal levels across animals to give an overall density. These densities were represented using a colour-coded scale, onto mouse brain atlas sections using Adobe Illustrator CS5. Sections throughout the pons and midbrain were also mapped and more rostral sections were examined for the presence of puncta.

### Statistical analysis

Data are expressed as means ± standard error of the mean. Data sets were analysed for statistical significance using Prism 5 (GraphPad Software, San Diego, CA). The normality of data was assessed using the D’Agostino-Pearson test and parametric or non-parametric testing chosen accordingly. Student’s paired t-tests, Wilcoxon matched pairs signed ranks test (both two-tailed) or Friedman tests with Dunn’s post-tests were used to determine statistical differences for WHBP data. Two-way repeated measures ANOVAs were used to analyse behavioural data, followed by Bonferroni post-tests. Differences were considered significant at p<0.05.

### Materials

All salts and drugs were from Sigma unless otherwise stated. Naloxone hydrochloride (Tocris, Bristol, UK; stock solution 2mM in dH_2_O) and SHU9119 (Bachem, Weil am Rheim, Germany; stock solution 186μM in PBS) were administered systemically by addition to the circulating perfusate.

## Results

**Fig 1:**
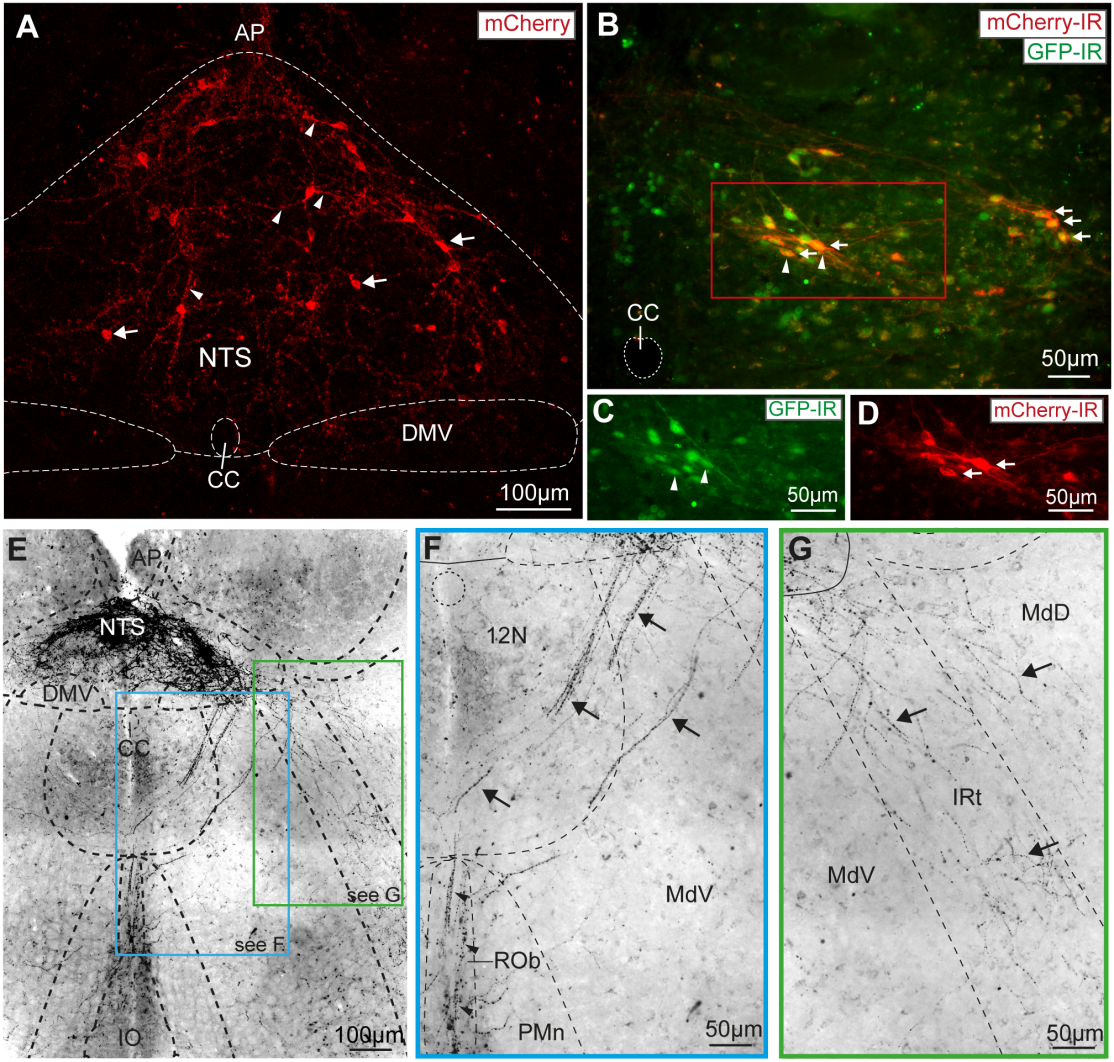
Vector mediated transduction of NTS_POMC_ neurons. (A) Native mCherry fluorescence in AAV-EF1α-DIO-ChR2-mCherry transduced neurons (arrows) and processes (arrowheads) in the NTS. (Confocal z-stack, 25μm stack in 1μm increments). (B) Vector transduction of NTS_POMC_ neurons was confirmed by co-localisation of mCherry (arrows) and GFP (arrowheads) expression in POMC-Cre-GFP mice. Double immunohistochemistry for GFP (C) and mCherry (D) showed 88% of transduced neurons were positive for GFP (Bregma -7.64mm). (E) Varicose axonal projections from NTS_POMC_ neurons to ventral regions of the caudal medulla were revealed by immunocytochemistry for mCherry (shown in inverted grayscale for clarity and also in insets F, G; Bregma -7.76mm). (F) NTS_POMC_ neuronal axons travelled ventromedially without branching through the edge of the hypoglossal nucleus (arrows) to form a terminal field in the raphe obscurus nucleus (arrowheads). (G) A second bundle of NTS_POMC_ neuronal axons travelled ventrolaterally (arrows) through the medullary and intermediate reticular nucleus to the ventral medulla. AP, area postrema; CC, central canal; DMV, dorsal motor nucleus of the vagus; IO, inferior olive; IRt, intermediate reticular nucleus; MdD, medullary reticular nucleus (dorsal); MdV, medullary reticular nucleus (ventral); PMn, paramedian reticular nucleus; ROb, raphe obscurus; 12N, hypoglossal nucleus.

To selectively express Cre-recombinase and enhanced green fluorescent protein (EGFP) in POMC neurons (POMC-Cre-GFP mice) we crossed POMC-Cre mice [27] with reporter mice (Tg(Gt(ROSA)26Sor-EGFP)). The fluorescent neurons in this POMC-Cre-GFP mouse line have previously been shown by *in situ* hybridisation to express mRNA for POMC in both the ARC and the NTS (see Allen Mouse Brain Atlas, http://tinyurl.com/Allen-Brain-Atlas-POMC-NTS). To allow opto-genetic activation of NTS_POMC_ neurons we expressed a channelrhodopsin-2 (ChR2)-mCherry fusion protein by unilateral stereotaxic microinjection of a Cre-dependent adeno-associated viral vector (AAV-EF1α-DIO-ChR2-mCherry) [34]. Subsequent post-hoc histological analysis showed native mCherry fluorescence in the somata and processes of a discrete subgroup of NTS neurons (Fig 1A, 3–4 weeks after injection). The average number of transduced neurons was 234±39 per animal (n = 6). The mCherry expression was co-localised with GFP indicating successful transduction of NTS_POMC_ neurons (Fig 1B–1D). Almost all of the mCherry-labelled neurons (95%, n = 6) were found within the NTS caudal to obex (from bregma -7.48 to -8.00mm) being distributed preferentially in the commissural, medial, ventral, ventrolateral and dorsolateral subnuclei—regions of the NTS known to be involved in nociceptive and cardiorespiratory reflex control [24, 25].

### NTS_POMC_ neurons have projection targets throughout the brainstem

**Fig 2:**
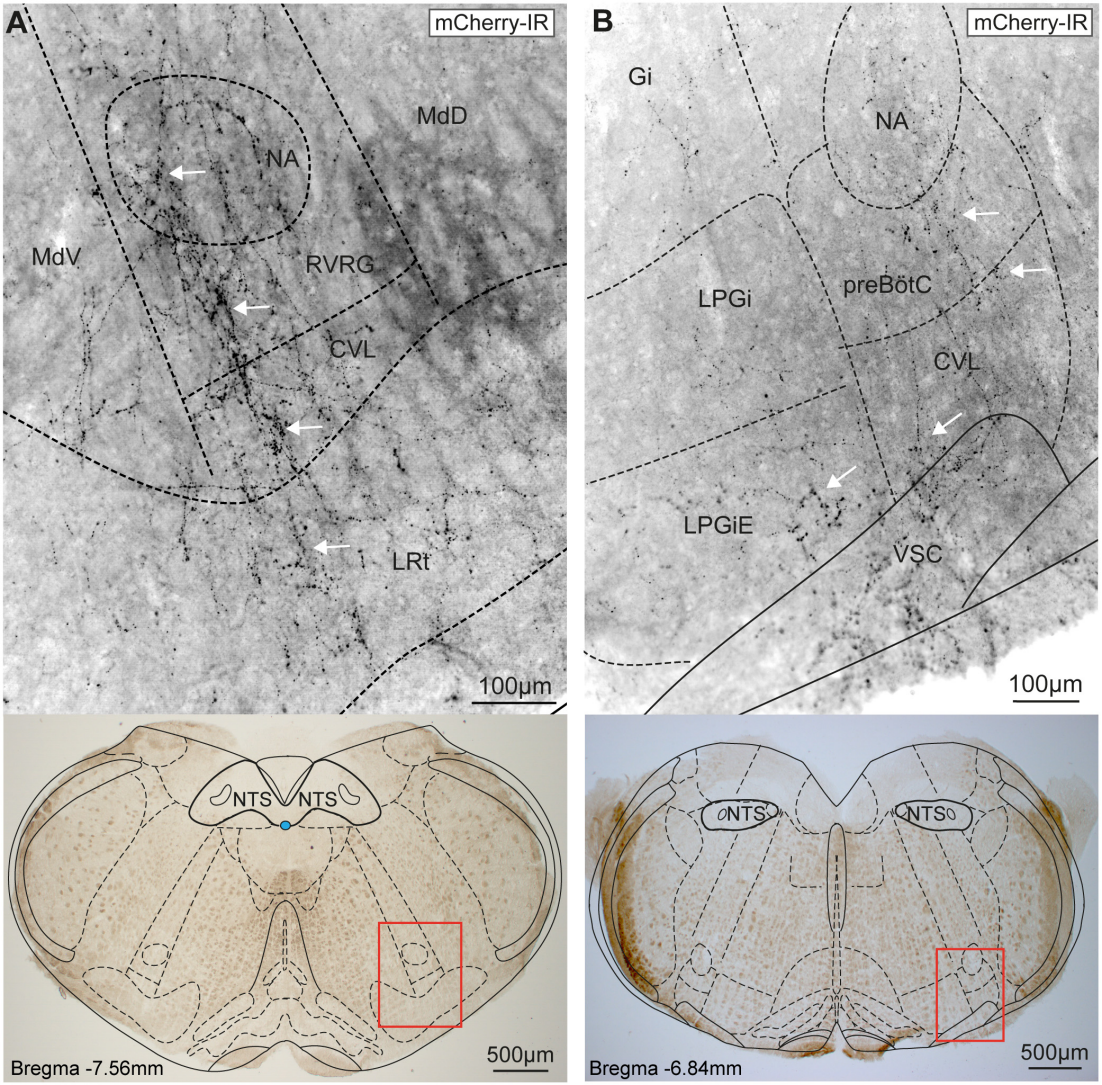
Axons from NTS_POMC_ neurons target the ventrolateral medulla. (A) Fine varicose axons outlined with ChR2-mCherry from AAV-EF1α-DIO-ChR2-mCherry transduced NTS_POMC_ neurons were found in the caudal ventrolateral medulla targeted to NA, RVRG, CVL and LRt (white arrows) but were largely absent from the reticular nuclei. (B) In the rostral medulla the axons were also targeted to specific regions including NA, preBötC, CVL and LPGiE (arrows). Imaged area shown below as red rectangle on brightfield images. Axonal fibres revealed by fluorescence immunocytochemistry for mCherry and shown in inverted grayscale for clarity. CVL, caudo-ventrolateral reticular nucleus; Gi, gigantocellular reticular nucleus; LPGi(E), lateral paragigantocellular nucleus (external part); LRt, lateral reticular nucleus; MdD, medullary reticular nucleus (dorsal); MdV, medullary reticular nucleus (ventral); NA, nucleus ambiguus; pre-BötC, pre-Bötzinger Complex; RVRG, rostral ventral respiratory group; VSC, ventral spinocerebellar tract.

To identify the projection targets of the NTS_POMC_ neurons we took advantage of the propensity of the ChR2-mCherry fusion protein to outline the axonal processes of the transduced neurons. The NTS_POMC_ neurons extended varicose axonal projections from the NTS ventrally into the medulla. The fine terminal axons were demonstrated within the distal target areas by immunohistochemical amplification of mCherry fluorescence. In the coronal plane, the axons travelled along two distinct tracks from the NTS (Fig 1E); each emerging from the lateral NTS with streams of fibres projecting, without branching, ventrolaterally and ventromedially. The axons coursing ventromedially projected through the medullary reticular nucleus and hypoglossal nucleus, heading ventrally along the midline to the nucleus raphe obscurus (Fig 1F). The ventrolaterally-directed axons projected through the medullary and intermediate reticular nuclei towards the ventrolateral medulla (VLM, Fig 1G). These axons within the VLM (n = 4 mice) were distributed as a network of branching terminal fibres extending throughout the nucleus ambiguus (NA), rostral ventral respiratory group, caudo-ventrolateral reticular nucleus, lateral reticular nucleus, pre-Bötzinger complex and lateral paragigantocellular nucleus (Fig 2). The fibres in the VLM were densest in the caudal medulla (bregma -7.48mm to -7.92mm) at a similar rostrocaudal level in the medulla to the POMC somata within the NTS but were also found in more rostral sections (up to the caudal end of the facial nucleus, bregma -6.5mm). The presence of fine axonal branches in these key brainstem cardiorespiratory and nociceptive control regions raised the question of whether the NTS_POMC_ neurons were making synaptic contacts.

**Fig 3:**
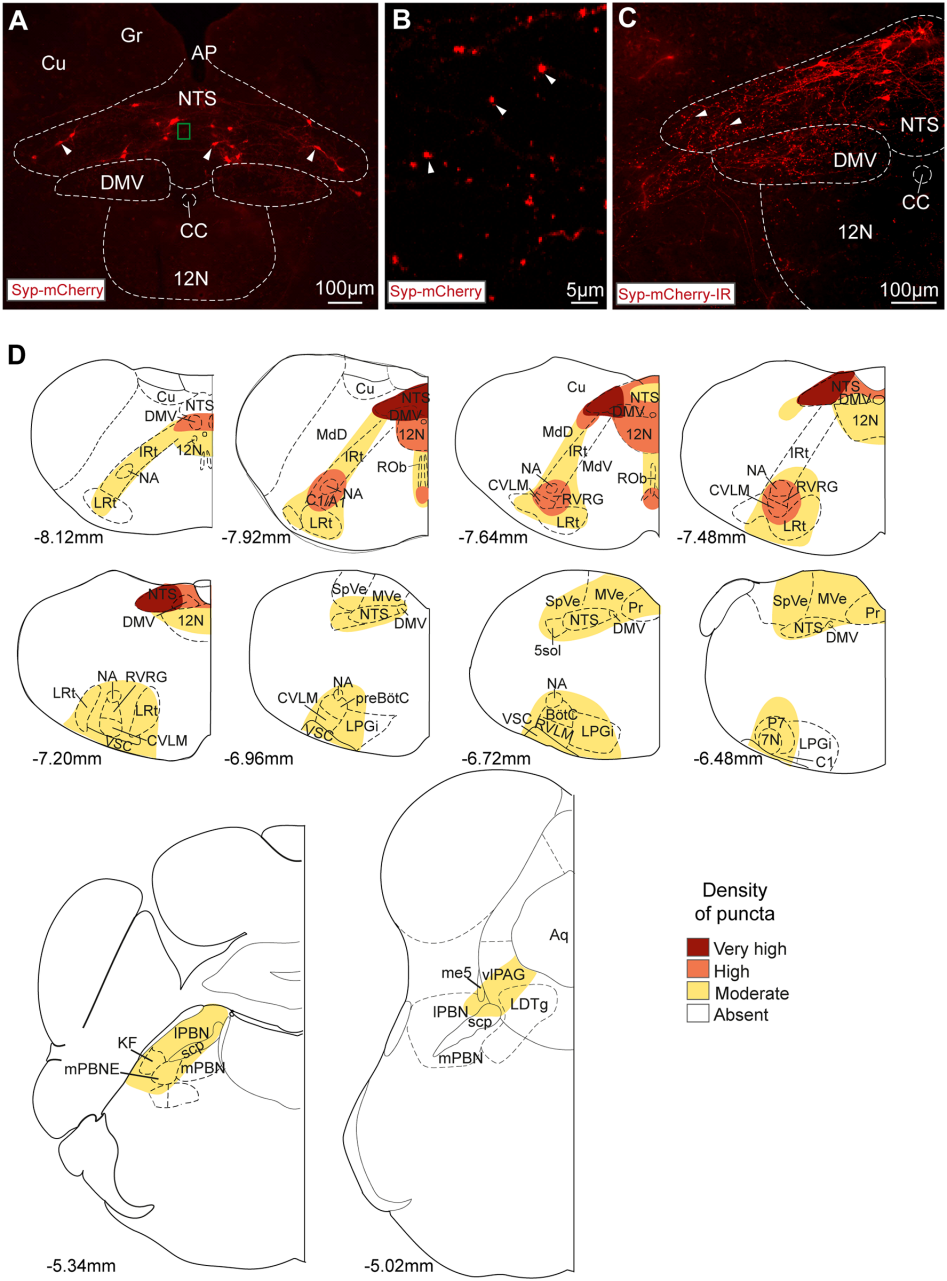
NTS_POMC_ neuronal terminals are targeted to specific brainstem territories. (A) Syp-mCherry fluorescence (native) in AAV-hSyn-FLEX-Synaptophysin-mCherry transduced neurons (arrowheads) in the NTS. (B) Syp-mCherry filled fluorescent puncta (arrowheads, ~0.5–1μm in diameter) were noted throughout the NTS. Area corresponds to small green box in (A), confocal z-stack maximal intensity projection (20 x 1μm slices). (C) A higher density of puncta was seen after immunohistochemistry to amplify Syp-mCherry fluorescence in the NTS (arrowheads) and in the dorsal motor nucleus of the vagus. (D) Distribution of Syp-mCherry-IR puncta from NTS_POMC_ neurons in the medulla, pons and midbrain. The relative densities of puncta across animals (n = 3) are shown mapped onto atlas sections (Franklin & Paxinos, 2007, distance from bregma indicated). 7N, facial nucleus; 12N, hypoglossal nucleus; A1, noradrenergic group; AP, area postrema; Aq, aqueduct; BötC; Bötzinger complex; C1, adrenergic group; CC, central canal; Cu, cuneate nucleus; CVLM, caudal ventrolateral medulla; DMV, dorsal motor nucleus of the vagus; Gr, gracile nucleus; IO, inferior olive; IRt, intermediate reticular nucleus; KF, Kölliker-Fuse nucleus; LDTg, laterodorsal tegmental nucleus; lPBN, lateral parabrachial nucleus; LPGi, lateral paragigantocellular nucleus; LRt, lateral reticular nucleus; MdD/V, medullary reticular nucleus (dorsal/ventral part); me5, mesencephalic trigeminal tract; mPBN, medial parabrachial nucleus; mPBNE, medial parabrachial nucleus (external part); MVe, medial vestibular nucleus; NA, nucleus ambiguus; ROb, raphe obscurus nucleus; P7, perifacial zone; preBötC, pre-Bötzinger complex; Pr, prepositus nucleus; RVLM, rostral ventrolateral medulla; RVRG, rostral ventral respiratory group; scp, superior cerebellar peduncle; SpVe, spinal vestibular nucleus; VSC, ventral spinocerebellar tract; vlPAG, ventrolateral periaqueductal gray.

To examine whether this distribution of axonal fibres actually reflected the location of synaptic terminals of NTS_POMC_ neurons, we stereotaxically microinjected a Cre-dependent AAV vector to express synaptophysin-mCherry fusion protein (AAV-hSyn-FLEX-Syp-mCherry) into the NTS of POMC-Cre-GFP mice. This produced a similar pattern of expression of Syp-mCherry (as had previously been seen with the ChR2-mCherry vector) in the somata of a discrete subset of neurons at the injection site (Fig 3A, 264±13, n = 4). The majority of these Syp-mCherry-labelled somata were found in the NTS (87%) in sections caudal to obex.

Numerous Syp-mCherry-containing puncta (~0.5–1μm in diameter) were visible within the NTS (Fig 3B) and brainstem. These puncta had a consistent distribution across mice (n = 4) and were concentrated in the same brainstem territories that contained the projection fibres of the NTS_POMC_ neurons (as documented above). The distribution and comparative densities of NTS_POMC_ neuronal puncta is summarised in Fig 3D (n = 3). The highest densities of puncta were found in the caudal NTS close to the injection site: specifically in the commissural, ventrolateral, ventral, medial, dorsolateral, dorsomedial and intermediate subnuclei and in the underlying dorsal vagal motor nucleus (DMV, Fig 3C). Puncta were found at moderate density throughout the rest of the rostro-caudal extent of the NTS and DMV (Fig 3D). High densities of puncta were also found in the medulla outside the vagal complex in the VLM caudal to obex, including the NA, rostral ventral respiratory group and caudal ventrolateral medulla and also in the hypoglossal nucleus. Moderate densities of puncta were noted in the raphe obscurus; caudal part of the lateral paragigantocellular nucleus; rostral ventrolateral medulla; and in the region of the pre-Bötzinger and Bötzinger complexes (Fig 3D, n = 3).

More rostrally in the pons and midbrain, a moderate density of puncta was found in the ventrolateral periaqueductal gray (vlPAG); lateral parabrachial nucleus (lPBN); Kölliker-Fuse nucleus and external part of the medial parabrachial nucleus (Fig 3D, n = 3). This indicates that in addition to ventrolateral and ventromedial tracts of NTS_POMC_ neuronal fibres, there is also a rostral tract of POMC neuronal fibres that project from the NTS to the dorsal pons and midbrain. Examination of brain territories ranging from the midbrain (bregma -4.60mm) through to the frontal lobes (bregma +2.80mm) did not reveal any further puncta.

### Activation of NTS_POMC_ neurons evokes bradycardia, augments respiratory sinus arrhythmia and causes bradypnoea

**Fig 4:**
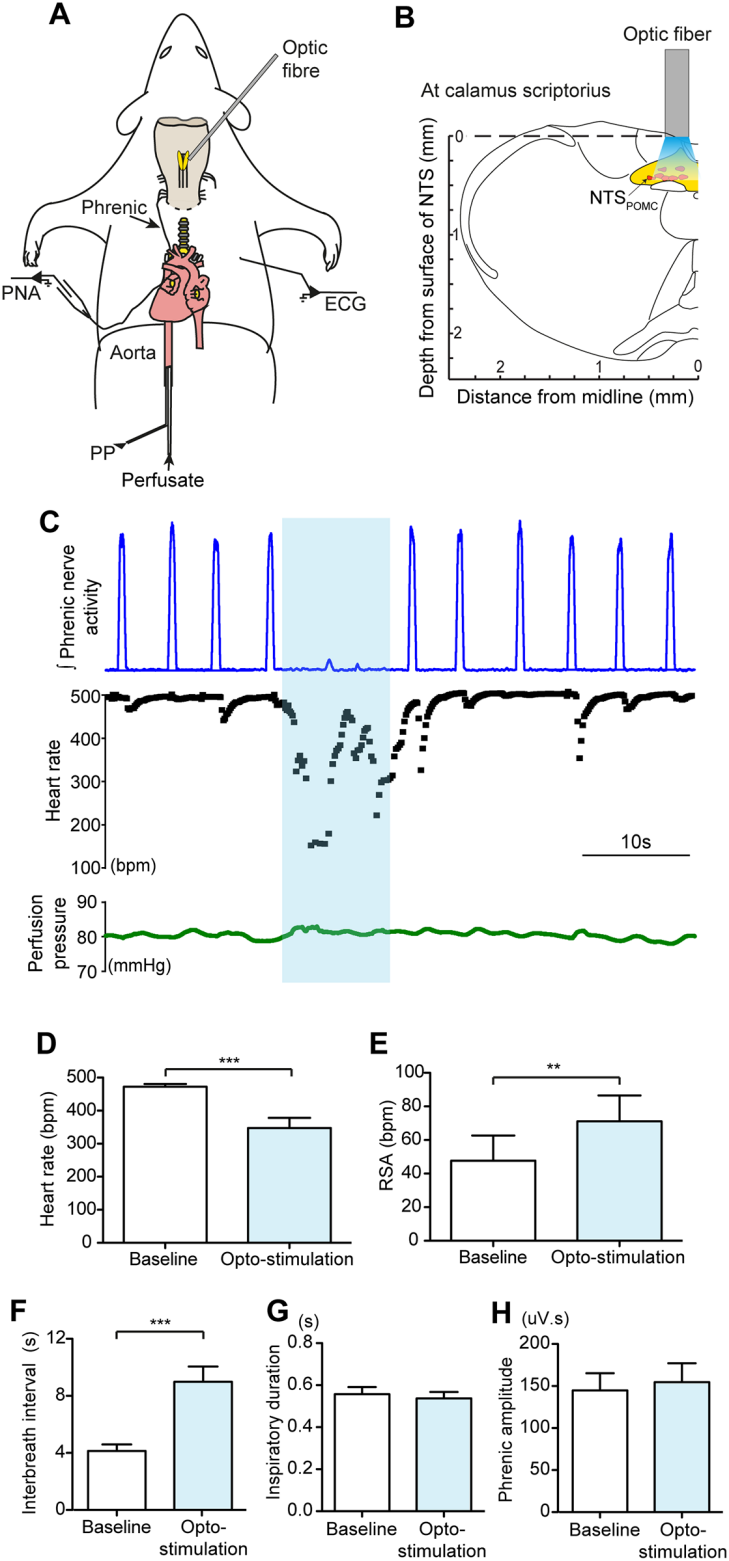
Opto-stimulation of NTS_POMC_ neurons in the WHBP evokes stereotyped cardio-respiratory responses. (A) Schematic diagram of the functional opto-stimulation experiment in WHBP. The mouse is decerebrated at the pre-collicular level and perfused via the descending aorta. Phrenic nerve activity (PNA) and electrocardiogram (ECG) are recorded and the perfusion pressure is monitored. The optic fiber was positioned on the dorsal surface of the medulla. (B) Coronal medullary slice schematically showing the optic fiber stimulation site (bregma -7.76mm). (C) Opto-stimulation of the NTS_POMC_ neurons for 10s (10Hz, 4.6mW; blue bar) evoked an apnoea, bradycardia and increased RSA. (D-F) Group data showing the responses to opto-stimulation (10Hz, 4.6mW for 10s) of NTS_POMC_ neurons consisting of: (D) bradycardia (n = 21, paired t-test); (E) increased RSA (n = 20, Wilcoxon test) and (F) bradypnoea (n = 21, Wilcoxon test) without any significant change in either the duration of (G) inspiration or (H) phrenic burst amplitude (n = 14, paired t-test). Data are represented as mean ± SEM. ** p<0.01, *** p<0.001. PNA, phrenic nerve activity; PP, perfusion pressure.

The pattern of NTS_POMC_ neuronal projections to key cardiorespiratory centres such as the NA, ventral respiratory group and the raphe suggested a role in autonomic regulation alongside the hypothesised role in the regulation of pain. We therefore used the working heart brainstem preparation (WHBP) to examine the effect of opto-stimulation of NTS_POMC_ neurons on cardiorespiratory control in mice that had received unilateral stereotaxic NTS microinjections of AAV-EF1α-DIO-ChR2-mCherry. The WHBP is an arterially perfused decerebrate preparation [32] facilitating access to the brainstem (without the confound of anaesthesia) while allowing simultaneous recording of ECG, phrenic nerve activity and perfusion pressure (Fig 4A). NTS_POMC_ neurons were activated by illumination from an optic fibre placed on the dorsal surface of the brainstem at the rostrocaudal level of *calamus scriptorius* (200μm lateral to midline), immediately above the vector injection site (Fig 4B).

Opto-stimulation (using a standardised stimulus—10Hz, 4.6mW for 10s) produced stereotyped cardiorespiratory responses, consisting of a bradycardia and transient apnoea (Fig 4C). The amplitude of RSA (the vagally-mediated, respiratory related fluctuation in heart rate) was also increased by opto-stimulation. These responses were time locked to the stimulus, had a short latency to onset and each recovered to baseline when opto-stimulation ended. Group analysis of the response to this pattern of opto-stimulation showed a bradycardia (amplitude = 125±26 beats per minute, bpm); from a baseline of 472±9 bpm to 348±31 bpm (p = 0.0001, n = 21 mice, paired t-test; Fig 4D). The latency to onset of the bradycardia was 1.1±0.2s (n = 21), and it lasted for the period of opto-stimulation. RSA was augmented by opto-stimulation with an increase in peak-to-trough amplitude from 47±15 to 71±15 bpm (p = 0.0012, n = 20, Wilcoxon signed rank test; Fig 4E). Opto-stimulation also produced a transient apnoea with slowing of the respiratory rate. The interbreath interval increased from 4.1±0.5s to 9.0±1.1s during stimulation (p<0.0001, n = 21, Wilcoxon signed rank test; Fig 4F) with no significant change in the amplitude of the phrenic nerve activity or the duration of inspiration (Fig 4G and 4H).

**Fig 5:**
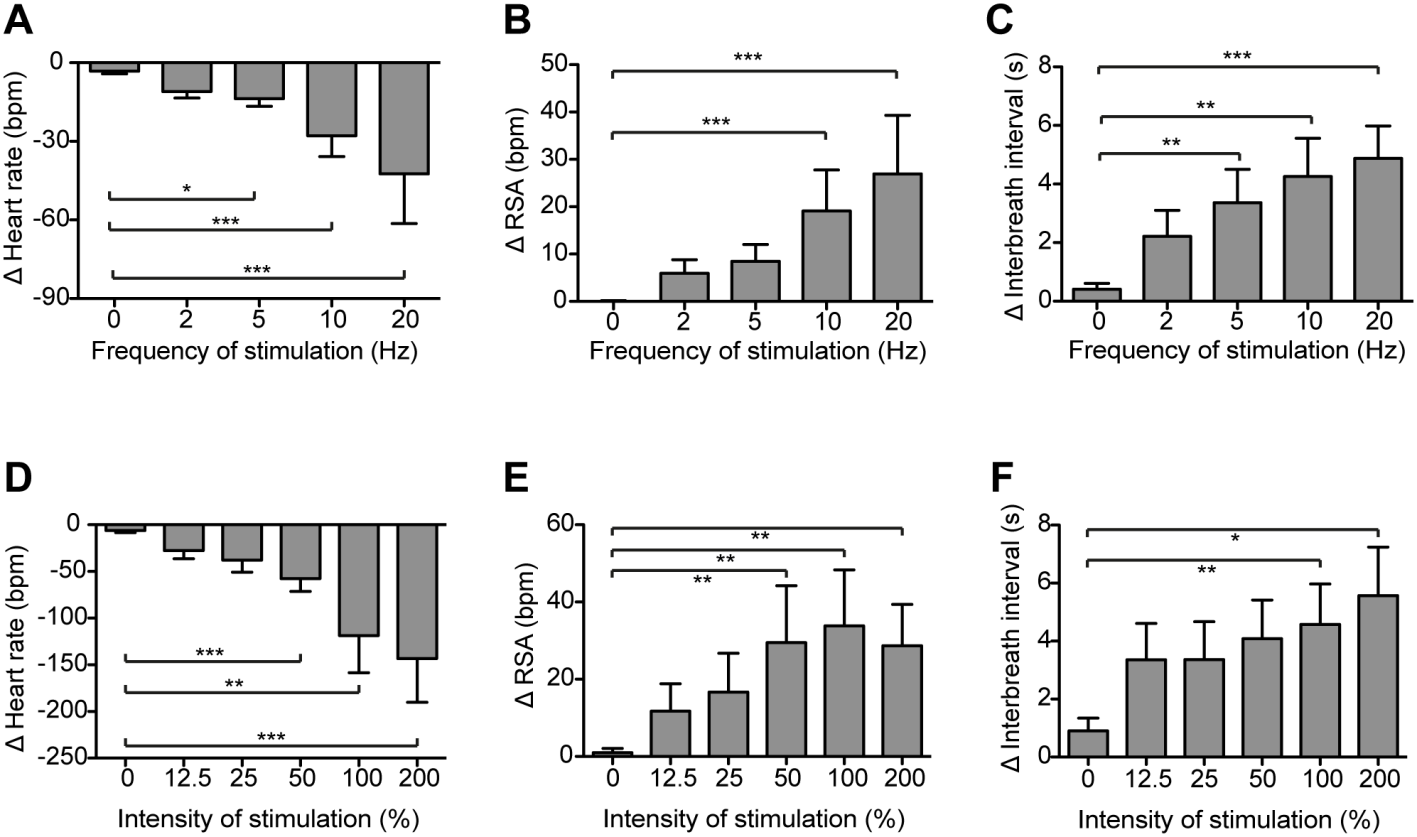
Frequency- and intensity-dependence of cardiorespiratory responses to NTS_POMC_ opto-stimulation. The cardiorespiratory response to NTS_POMC_ opto-stimulation (4.6mW for 10s) was frequency-dependent: (A) bradycardia (n = 9); (B) RSA (n = 10); and (C) interbreath interval (n = 10). Similarly the cardiorespiratory response to opto-stimulation (10Hz for 10s) was dependent upon stimulus intensity (100% = 4.6mW): (D) bradycardia (n = 8); (E) RSA (n = 7); and (F) interbreath interval (n = 8). Data are shown as change from baseline, mean ± SEM; * p<0.05, ** p<0.01 *** p<0.001. Friedman tests with Dunn’s post-hoc analysis.

All cardiorespiratory responses were reproducible on repeated opto-stimulation without evidence of desensitisation and their duration could be prolonged if the period of stimulation was extended (e.g. 1 min). These cardiorespiratory responses were all dependent on the frequency (2-20Hz) and intensity of stimulation, with higher frequencies and intensities of opto-stimulation producing a larger bradycardia, longer duration of apnoea and an increase in RSA (Fig 5). The effects of opto-stimulation were only obtained from illumination sites over the caudal region of the NTS and progressively diminished when the optic fibre was moved rostrally.

### Cardiorespiratory responses to opto-stimulation of NTS_POMC_ neurons are opioid-mediated

**Fig 6:**
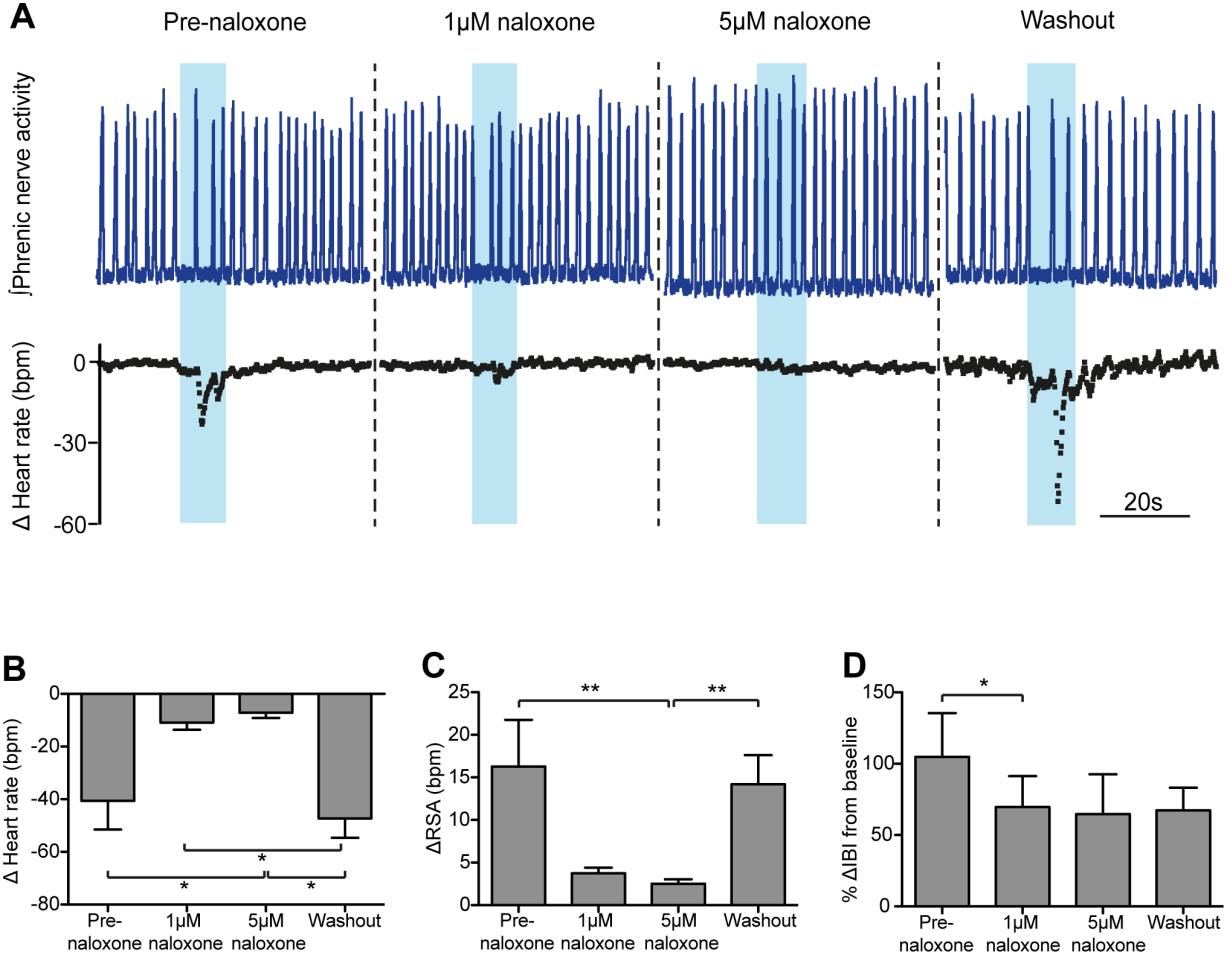
Systemic naloxone attenuates the cardiorespiratory responses to opto-stimulation of NTS_POMC_ neurons. (A) Opto-stimulation of the NTS_POMC_ neurons in the WHBP (10Hz for 10s) evoked the characteristic bradycardia, transient apnoea and increased RSA. This response was blocked by naloxone (1μM and 5μM, added to the perfusate). Following washout of naloxone (10mins), the cardiorespiratory responses to opto-stimulation recovered. Grouped data show that systemic naloxone at 1μM and 5μM attenuated the: (B) bradycardia, (C) change in RSA, and (D) bradypnoea (percentage change in inter-breath interval, %ΔIBI), in response to opto-stimulation. Data are represented as mean ± SEM. Friedman tests with Dunn’s post tests (n = 6). *<0.05, ** p<0.01.

POMC neurons are thought to signal through the release of the peptide cleavage products of POMC, potentially including β-endorphin and/or melanocortins. Therefore, to test the hypothesis that the cardiorespiratory responses to activation of NTS_POMC_ neurons are mediated by an opioid, the antagonist naloxone was added to the circulating perfusate. The opto-stimulation-evoked bradycardia, increased RSA and bradypnoea were all diminished in the presence of naloxone (1μM and 5μM, Fig 6A).

**Fig 7:**
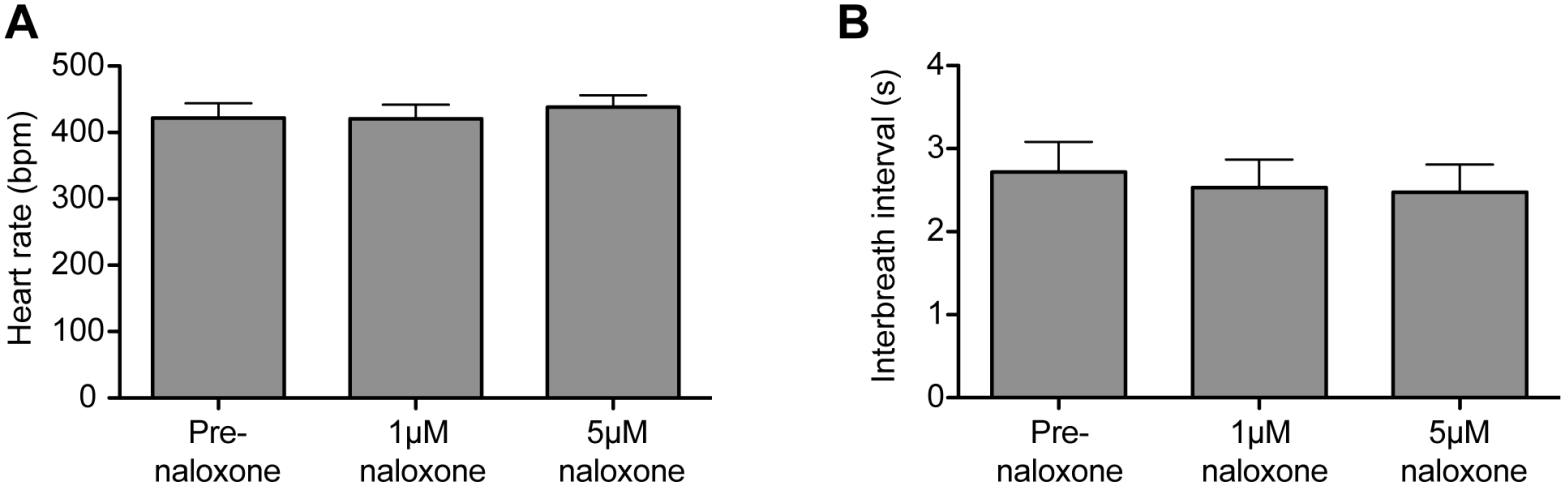
Naloxone has no effect on heart rate or respiratory frequency. Administration of naloxone (1 and 5μm) to the perfusate in the WHBP had no effect on either baseline: (A) heart rate or (B) on respiratory frequency (n = 6 preparations). Friedman tests with Dunn’s post-hoc analysis for naloxone vs baseline.

Naloxone (at both 1 and 5μM) attenuated the bradycardia (from -40.6±10.9 to -7.2±1.9 bpm (5μm), p<0.05; Fig 6B), which recovered following washout (to -47.2±7.4 bpm; p<0.05, 5μM naloxone vs. washout; Friedman test (p = 0.0001) with Dunn’s post tests for each condition; n = 6). Naloxone also reduced the augmentation of RSA by opto-stimulation (Fig 6C) from 16.3±5.5 pre-naloxone to 3.7±0.7 bpm in the presence of the opioid antagonist (1μM). Increasing the dose of naloxone to 5μM further attenuated the RSA response (to 2.5±0.5 bpm, p<0.01 vs baseline) and the opto-stimulation-evoked augmentation of RSA returned following washout of naloxone (to 14.2±3.4 bpm; p<0.01; Friedman test (p<0.0001) with Dunn’s post tests; n = 6). Additionally, naloxone reduced the bradypnoeic effect of opto-stimulation (Fig 6D). Opto-stimulation induced a 105±31% increase in interbreath interval, which was significantly reduced by naloxone (1μM) (70±22%; p<0.05, Friedman test (p = 0.043) with Dunn’s post test; n = 6). 5μM naloxone did not further reduce the change in interbreath interval and the respiratory response did not recover after 30 minutes of naloxone washout. Naloxone alone had no significant effect on either baseline heart rate or respiratory frequency (Fig 7, n = 6).

**Fig 8:**
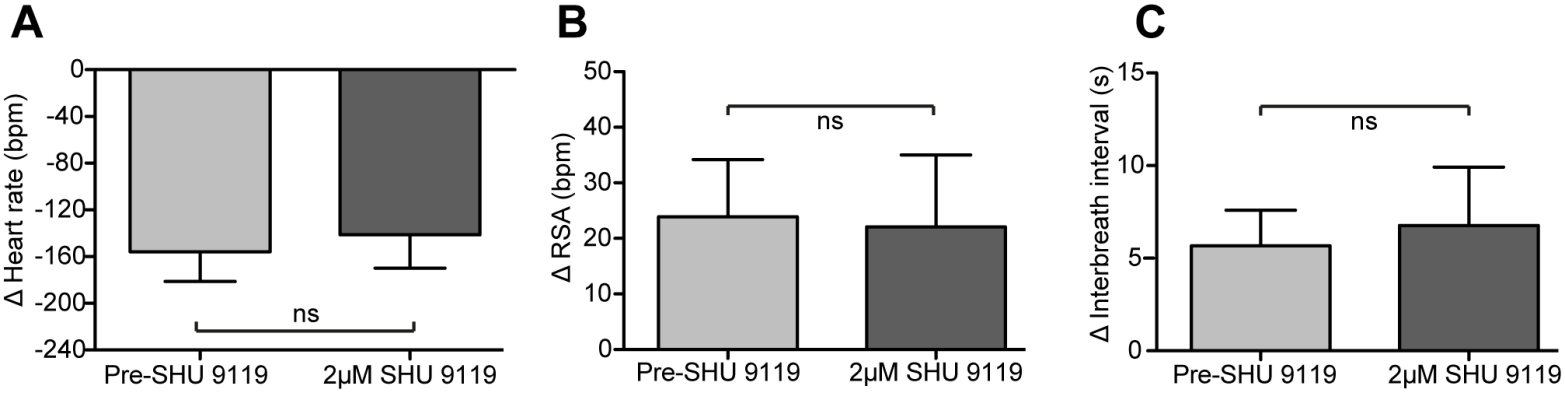
The melanocortin receptor antagonist SHU9119 had no effect on the cardiorespiratory responses to opto-stimulation. Systemic administration of SHU9119 (2μM) had no effect on the (A) bradycardia, (B) RSA responses, and (C) bradypnoeic responses to opto-stimulation of NTS_POMC_ neurons in the WHBP. Data are represented as mean±SEM. Wilcoxon matched-pairs signed rank tests (n = 7).

The reversible block of the opto-stimulation-evoked cardiorespiratory responses by naloxone supports a role for an endogenous opioid peptide in the mediation of these responses. However, given that α-MSH has been found in cell bodies in the NTS [35], we examined the effect of SHU9119, an antagonist of MC3 and MC4 receptors—the main melanocortin receptors expressed in the brain [36], on the evoked cardiorespiratory responses. The dose of SHU9119 was chosen based on prior *in vitro* brainstem slice experiments that blocked melanocortin agonist effects in the NTS (1μM SHU9119, [37]) and dorsal motor nucleus of the vagus (0.5μM SHU9119, [38]). The addition of SHU9119 (2μM) to the perfusate had no effect on the cardiorespiratory responses to opto-stimulation of NTS_POMC_ neurons (Fig 8, n = 7). These data indicate that the cardiorespiratory responses to NTS_POMC_ opto-stimulation are not mediated by α-MSH.

### POMC neurons project to vagal preganglionic neurons in the nucleus ambiguus

**Fig 9:**
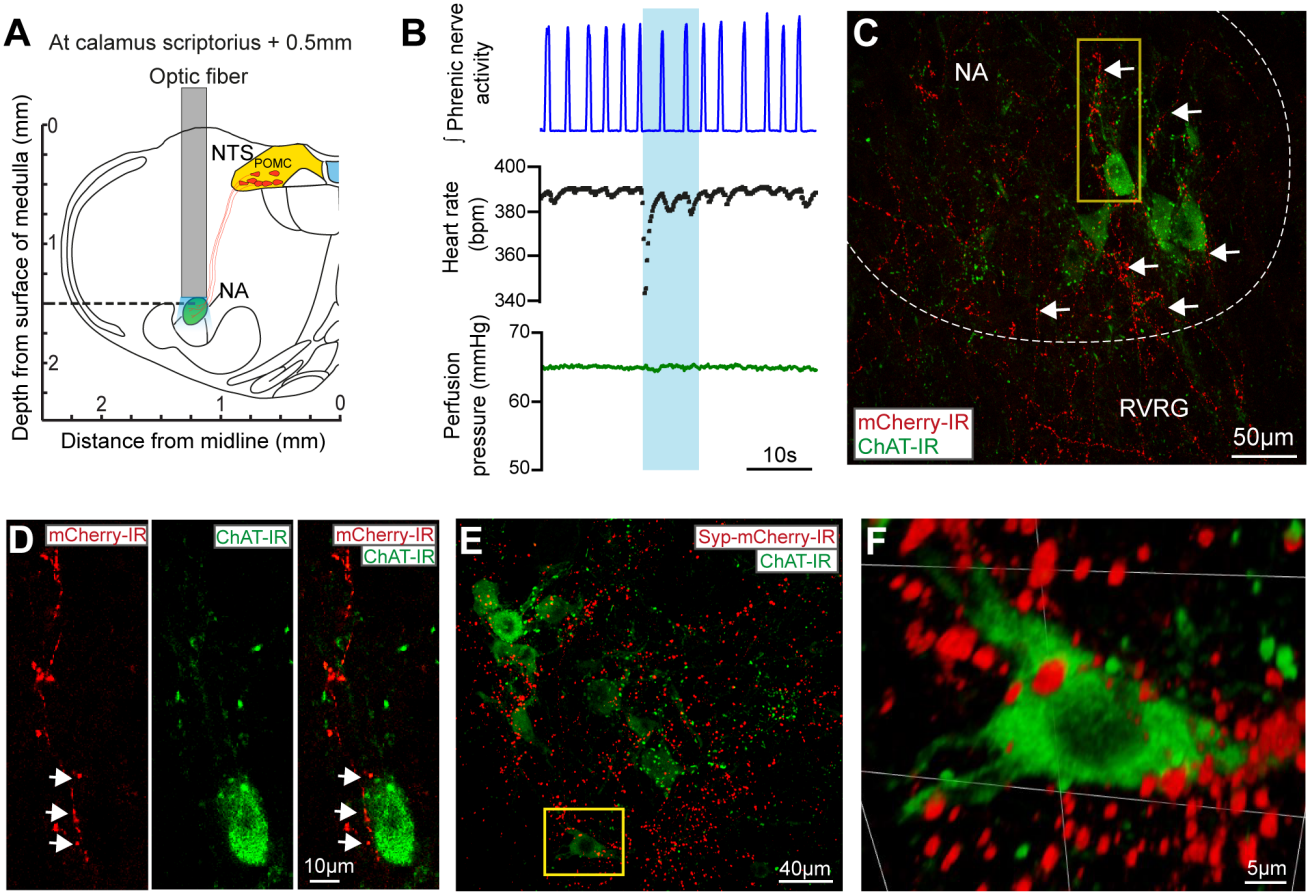
NTS_POMC_ neuronal projections to the nucleus ambiguus may mediate the bradycardic response to opto-stimulation. Opto-stimulation was performed over the NA in the WHBP after prior transduction of NTS_POMC_ neurons with AAV-EF1α-DIO-ChR2-mCherry. (A) Schematic diagram of the position of the optic fiber over the NA (0.5mm rostral and 1.25mm lateral to calamus scriptorius; 1.5mm deep to the dorsal surface of the medulla). (B) Opto-stimulation (10Hz, 4.6mW for 10s) over the region of NA produced a bradycardia and modest bradypnoea. (C) NTS_POMC_ neuronal axons labeled with ChR2-mCherry were found in the vicinity of cholinergic vagal preganglionic neurons in the NA (merged z-stack: 15μm stack in 1μm steps). (D) Inset of C, a single section (1μm) showing NTS_POMC_ axons (red) in the near vicinity of a cholinergic vagal preganglionic neuron (green). (E) Labelling with Syp-mCherry-IR showed NTS_POMC_ terminal puncta (merged z-stack: 17μm in 0.5μm steps) in the near vicinity of NA cholinergic neurons. (F) Inset of E, 3D visualisation of the z-stack, showing the presence of puncta surrounding an NA cholinergic neuron. NA, nucleus ambiguus; RVRG, rostral ventral respiratory group.

The NA is of particular interest because it contains the preganglionic cardiac vagal motor neurons (CVNs), located in the external formation [39, 40] and mediate the parasympathetic control of heart rate. We hypothesised that the bradycardia and augmentation of RSA seen on opto-stimulation may therefore be mediated by NTS_POMC_ neuronal projections to the NA. To investigate this, direct opto-stimulation over the NA was performed in preparations that had previously shown cardiorespiratory responses to opto-stimulation over the NTS. The optic fibre was then repositioned to sit above the NA in order to activate ChR2 in the terminals of NTS_POMC_ neurons (Fig 9A). In each case opto-stimulation over the NA (10Hz, 4.6mW) elicited bradycardia, accompanied by a transient apnoea or slowed respiratory frequency (n = 3, Fig 9B).

These cardiorespiratory responses to opto-stimulation over the NA were supported by anatomical tracing experiments, which showed dense labelling of NTS_POMC_ neuronal fibres (Fig 9C) and puncta (Fig 9E) in the NA. Both ChR2-mCherry axons and Syp-mCherry puncta in the NA were found most densely in sections caudal to obex and were seen to extend ventrolaterally outside of the compact, semi-compact and loose formations to the external formation of the NA, which is where the CVNs are found. NTS_POMC_ fibres and puncta were noted to be concentrated in the territory of NA cholinergic vagal preganglionic neurons (Fig 9D–9F). These combined anatomical and functional data suggests that POMC neurons project to the NA to modulate heart rate by vagal activation.

### Pharmaco-activation of NTS_POMC_ neurons is analgesic

To investigate whether NTS_POMC_ neurons also have a role in analgesia, we used a chemo-genetic approach [1] with expression of an engineered ion channel to allow targeted neuronal activation with a selective agonist. One group of mice received stereotaxic microinjections of the Cre-dependent vector AAV-hSyn-FLEX-PSAM-5HT_3_ (from the Sternson lab, see [1], PSAM group). This enabled expression of a modified α7-nicotinic acetylcholine receptor ligand binding domain fused to a 5-HT_3_ receptor ion pore (PSAM-5HT_3_). Nociceptive responses were examined in PSAM mice using the tail-flick assay (chosen for its known sensitivity to exogenous opioids [41–45] and to NTS stimulation [22, 46, 47]). Systemic administration of the selective PSAM receptor ligand, PSEM^89S^, was used to activate the NTS_POMC_ neurons.

**Fig 10:**
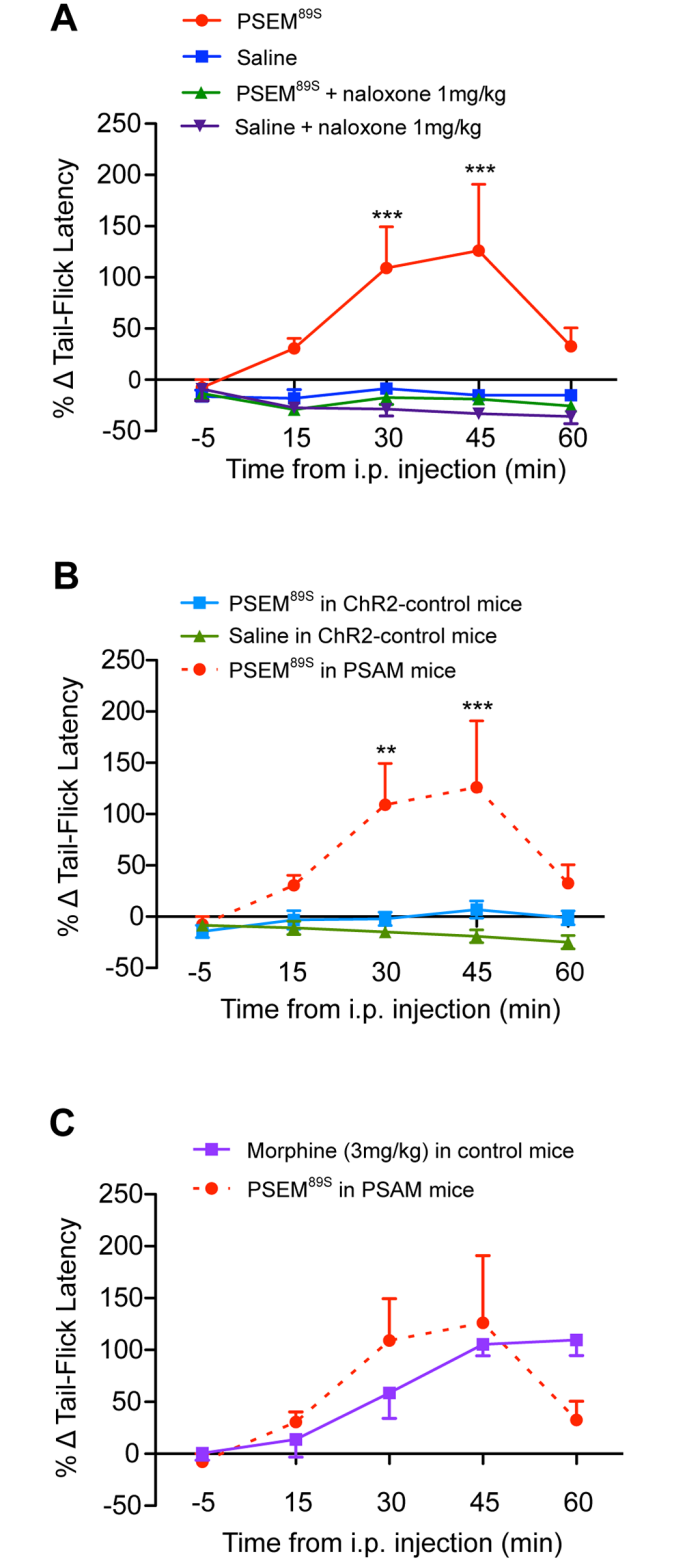
Chemo-activation of NTS_POMC_ neurons produces an opioid-mediated anti-nociceptive effect in the tail-flick assay. A. Tail-flick latencies were measured in POMC-Cre-GFP mice that had received NTS microinjections of AAV-hSyn-FLEX-PSAM-5HT_3_. Injection of the selective agonist PSEM^89S^ (90mg/kg) increased tail-flick latencies at 30 min and 45 min compared to saline. Pre-treatment with naloxone (1mg/kg, i.p) blocked the increase in tail-flick latencies evoked by PSEM^89S^. Two-way repeated measures ANOVA (p = 0.0002) with Bonferroni post-tests (***p<0.001) PSEM^89S^ vs saline and PSEM^89S^ vs Naloxone+PSEM^89S^ (n = 8). (B) Control experiment showing that PSEM^89S^ (90mg/kg) had no significant effect on tail-flick latencies in ChR2-control mice (POMC-Cre-GFP mice that had received NTS micro-injections of a control vector AAV-EF1α-DIO-ChR2-mCherry). The tail-flick latencies following PSEM^89S^ (90mg/kg) or saline administration in ChR2-control mice were not significantly different at any time point (Two way repeated measures ANOVA, n = 8). By comparison administration of PSEM^89S^ in PSAM mice (n = 8) significantly increased tail-flick latencies compared to those in ChR2-control mice at 30 and 45 min after injection. Two-way repeated measures ANOVA (p = 0.0035) with Bonferroni post-tests (**p<0.01, ***p<0.001, n = 8). (C) The analgesic effect of PSEM^89S^ in PSAM-mice (90mg/kg as in A) in the tail flick assay was comparable to that seen with morphine (3mg/kg, i.p.) administered to naïve mice of the same age (n = 6, CD1 mice). Data represented as % change in tail withdrawal latency from baseline; values are mean ± SEM.

Chemo-genetic activation of NTS_POMC_ neurons (PSEM^89S^, 90mg/kg, i.p.) exerted an antinociceptive action with an increase in withdrawal latency seen at 30 min (PSEM^89S^ = 110±40% vs saline = -9±5%; p<0.001) and at 45 min (PSEM^89S^ = 126±65% vs saline = -15±6%; p<0.001; n = 8 per group; 2-way repeated measures ANOVA (p = 0.0002) with Bonferroni post-tests; Fig 10A). The tail-flick latency had recovered to baseline levels by 60 min.

To investigate if the PSEM^89S^-evoked analgesia was opioid-mediated, naloxone (1mg/kg i.p.) was given 10 min prior to PSEM^89S^ injection. Pre-treatment with naloxone completely blocked the increase in tail-flick latency seen following injection of PSEM^89S^ (n = 8; Fig 10A). Naloxone only had an effect on tail-flick latencies in animals given PSEM^89S^ (as opposed to saline) and did not have a pro-nociceptive effect in its own right. These data suggest that the increased tail-flick latency following PSEM^89S^ injection is opioid-mediated and the magnitude of the PSEM^89S^ analgesia was equivalent to that produced by morphine (3mg/kg, i.p.) in naïve mice (Fig 10C).

Control experiments in POMC-Cre-GFP mice that had received NTS microinjections of a comparable control vector AAV-EF1α-DIO-ChR2-mCherry (ChR2-control mice) showed no change in tail flick latencies after PSEM^89S^ injection (90mg/kg) compared to saline injection (n = 8; Fig 10B). Taken together, these data suggest that the increased tail-flick latencies seen following PSEM^89S^ injection are due to specific activation of PSAM receptors expressed on the NTS_POMC_ neurons.

## Discussion

Using a combination of viral vector-mediated transduction of genetic actuators with Cre-recombinase targeting [27] we show that activation of the small cluster of POMC neurons (two to three hundred cells) in the NTS exerts strong effects on cardiorespiratory control and on nociceptive processing. These actions are all sensitive to naloxone indicating their mediation by an opioidergic mechanism, consistent with the release of β-endorphin from the POMC neurons. This identifies the NTS_POMC_ neurons as being a component of a neural circuit enabling the coordinated regulation of nociception and cardiorespiratory control.

**Fig 11:**
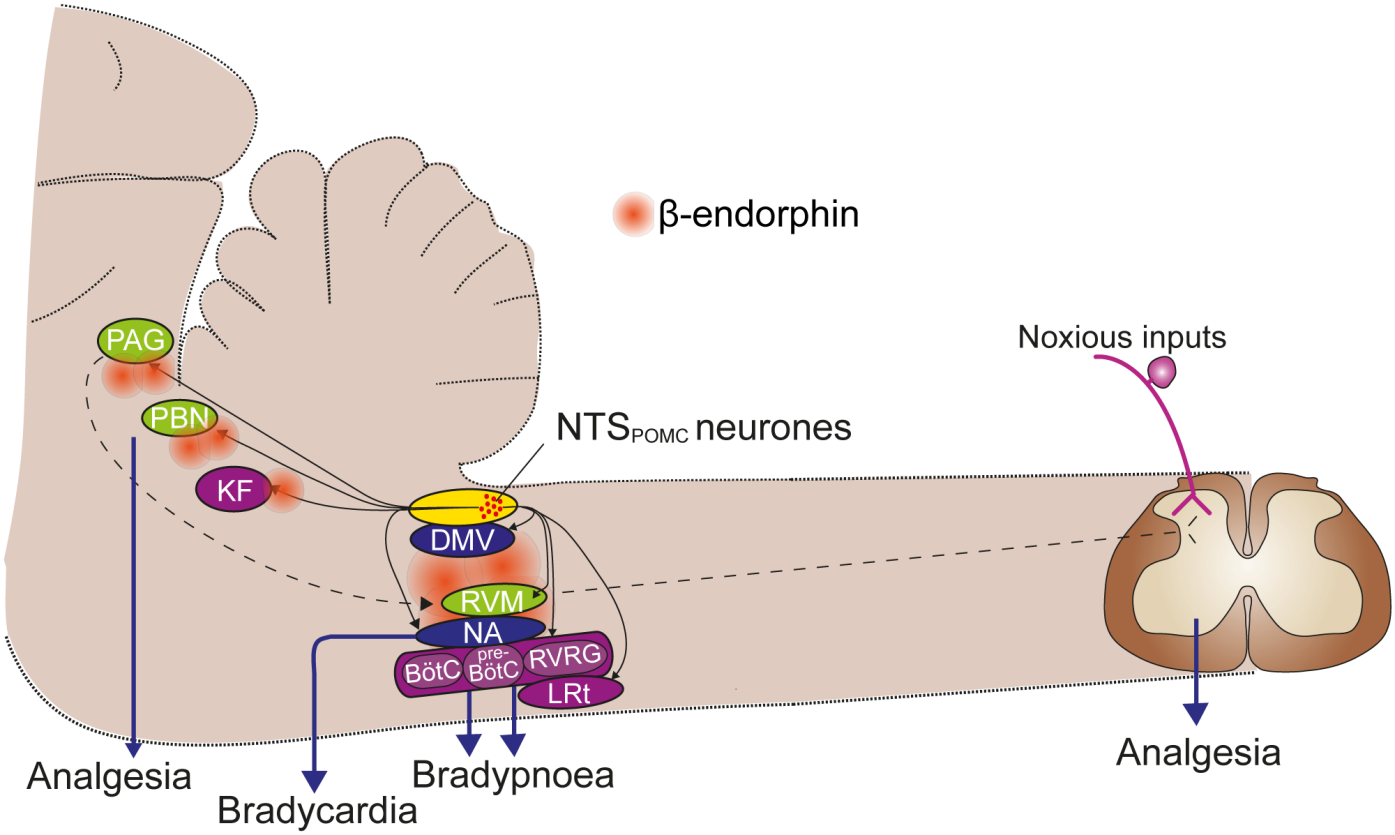
A schematic summary of the projections of NTS_POMC_ neurons and proposed sites for the functional effects. NTS_POMC_ neurons project selectively to multiple nuclei in the brainstem involved in cardiorespiratory and nociceptive control (black solid arrows). While several of these regions have multiple functions in autonomic, motor and sensory control (such as the PAG) they are grouped pragmatically by predominant action into vagal (blue), which are likely involved in the modulation of heart rate; respiratory (purple); and sensory, particularly nociceptive control (green). The nociceptive control may be mediated at either a brainstem level or by the recognised PAG-RVM-spinal cord pathway (dotted arrows). The sensitivity to opioid antagonists indicates that each of these pathways is likely to involve the release of β-endorphin. BötC, Bötzinger complex; DMV, dorsal motor nucleus of the vagus; KF, Kölliker-Fuse nucleus; LRt, lateral reticular nucleus; NA, nucleus ambiguus; PAG, periaqueductal gray; PBN, parabrachial nucleus; preBötC, preBötzinger complex; RVRG, rostral ventral respiratory group.

The NTS_POMC_ cells are projection neurons [15, 16, 48] and we show that they selectively target a number of important brainstem territories involved in cardiovascular, respiratory, autonomic and sensory control allowing them to potentially produce co-ordinated poly-modal regulation (Fig 11). These brainstem POMC neurons have been reported to be involved in signalling satiety [21, 49] but our findings extend their remit to encompass novel roles. Their activation engages a coordinated set of autonomic, motor and sensory changes. This composite response has many features common to those seen following the systemic administration of opioids, which include analgesia, respiratory slowing, bradycardia and increased RSA [3, 50–52].

Direct chemical or electrical stimulation of the NTS produces an analgesic effect [46, 53, 54] that is sensitive to opioid antagonists [10]. Similar analgesic effects are produced by stimulation of vagal nerve afferents in animals [55], in patients with vagal nerve stimulators (implanted for epilepsy) [23, 56] and in healthy volunteers following transcutaneous vagal stimulation [57]. Given that NTS_POMC_ neurons receive direct vagal afferent excitatory inputs [49], and their activation produces a naloxone-sensitive antinociceptive action, they form a credible circuit substrate for this stimulation-evoked analgesia.

This antinociceptive action could be generated by opioid release from POMC terminals in a number of brainstem sites, including at the level of the NTS itself, or the parabrachial nucleus to modulate ascending nociceptive information, or via descending influences to the spinal cord relayed via terminals in the PAG or the rostral ventromedial medulla (RVM) [8]. These observations raise the question of whether stress-evoked analgesia, which is sensitive to genetic ablation of β-endorphin [11] may also be mediated via the NTS_POMC_ neurons (as well as or instead of those in the ARC). The stress-analgesia circuit is known to involve structures such as the amygdala, hypothalamus and the PAG [7], which have all been recently shown by rabies trans-synaptic labelling to innervate the NTS_POMC_ neurons [48]. Thus the appropriate circuit connectivity exists allowing NTS_POMC_ neurons to potentially play a role in stress-induced analgesia. We also note that the PAG innervation from the NTS_POMC_ neurons appears to be predominantly to the ventrolateral column, which is intriguing as stimulation of that PAG territory produces an opioidergic analgesia accompanied by bradycardia and respiratory slowing [58]–all features seen on activation of the NTS_POMC_ neurons.

Activation of NTS_POMC_ neurons produced a rapid-onset bradycardia and an augmentation of RSA that was blocked by naloxone. This is likely to reflect an excitation of CVNs located in the NA [39, 40]. We have demonstrated that NTS_POMC_ neurons project to the NA and their terminals are found in close vicinity to cholinergic preganglionic neurons, which suggests that they could release β-endorphin onto the preganglionics. Such a projection of β-endorphin containing fibres from the NTS to the ventrolateral medulla, including the NA, has previously been demonstrated using immunocytochemistry and tract lesions [16]. We note, however, that the postsynaptic action of opioids is a canonical inhibition mediated by activation of potassium channels and closure of voltage gated calcium channels [3]. Indeed this effect has been documented for exogenous μ-opioid receptor agonists applied to CVNs *in vitro* [59], so this would not account for the excitation seen following NTS_POMC_ neuronal opto-stimulation. An alternative putative mechanism would be a presynaptic inhibition of GABA/glycinergic interneurons that leads to disinhibition of the vagal preganglionic neurons. This motif has been reported for opioid actions at a number of pain processing sites including the RVM and PAG [8, 53, 60]. As CVNs are known to have a tonic level of inhibition originating from nearby interneurons [61], which can be inhibited by the application of exogenous opioids *in vitro* [62, 63], then this opioid-mediated presynaptic inhibition would account for the increased vagal outflow. A previous opto-genetic study of hypothalamic POMC neurons demonstrated a comparable presynaptic inhibitory action on synaptic inputs to Agouti-related peptide neurons, mediated by an opioidergic mechanism [64]. It is apparent from our data that the bradycardia is phasically entrained to the respiratory drive, suggesting that it reflects an input from the respiratory pattern generator. However, the precise details of this mechanism, and its regulation by opioids, will require recordings from the local circuits in the ventral brainstem.

Opto-stimulation of NTS_POMC_ neurons produced a slowing of the respiratory rate, often with a period of apnoea, which was naloxone sensitive. This respiratory slowing was not accompanied by reductions in the amplitude or duration of inspiratory phrenic discharge (analogous to tidal volume). Thus this opioid-mediated action is similar to that seen with systemic administration of opioids in man; namely that an effect on frequency is seen before any reduction in tidal volume [52, 65]. The site of these effects of opioids on the central respiratory networks have been the focus of on-going debate [66, 67], and there is evidence that they can act within the pre-Bötzinger nucleus to produce a decrease in respiratory frequency [68], potentially by a postsynaptic inhibition of respiratory pattern generator neurons. We found that the axons and terminals of NTS_POMC_ neurons are found throughout much of the length of the ventral respiratory column indicating that they may be able to exert actions both within and outside the pre-Bötzinger complex. It may also be relevant that there was innervation within the pons to the region of the Kölliker-Fuse nucleus, which regulates respiratory pattern generation and is known to be opioid sensitive [69]. Preliminary evidence indicates that the Kölliker-Fuse nucleus may also be involved in the generation of RSA [70]. Such opto-genetic targeting of the NTS_POMC_ neurons may allow the role of these distinct territories to be tested by direct light activation over terminal fields, as we showed for the NA. Similarly the combination of selective opioid antagonists with the focal opto-activation may enable the specific receptor mechanisms mediating these actions to be determined.

Given that the NTS_POMC_ neurons receive direct inputs from visceral vagal afferents (Appleyard et al., 2005) and acutely signal satiety [21, 49] the findings of the current study raise the question of whether they could also be part of the circuit involved in feeding-associated (Mason and Foo, 2009) or sweet-taste analgesia [71], which are components of a hierarchical response that prioritises food acquisition over nocifensive behaviour. These responses have been explored in a number of different paradigms and there are some commonalities such as the involvement of the NTS (Anseloni et al., 2005), opioidergic mechanisms [72, 73] and an action via the RVM [72, 74]. Thus the NTS_POMC_ neurons could also have a role in feeding-associated analgesia as well as in stress-induced analgesia.

This small population of NTS_POMC_ neurons (200–300) can produce potent opioid-receptor-mediated changes in cardiorespiratory control and nocifensive behaviour that are similar to those seen with systemic opioid administration (equivalent to morphine 3mg/kg). These neurons may be part of endogenous analgesic circuits engaged in specific behavioural contexts to elicit a co-ordinated set of autonomic changes that produce a state of vagal predominance. It remains to be determined whether these neurons are indeed necessary for the expression of these behaviours and this will require targeted subtractive genetic manipulations to ablate or inhibit their function. A final question that merits consideration is whether NTS_POMC_ neurons constitute a homogenous population or whether there are functional sub-specialisations, even amongst a group of several hundred cells, as may be suggested by the presence of distinct, unbranching projection tracts that extend from the NTS through the brainstem to the diverse targets.

## Abbreviations

POMC: pro-opiomelanocortin
NTS: Nucleus of the solitary tract
ARC: Arcuate nucleus
α-MSH: alpha-melanocyte stimulating hormone
EGFP: enhanced green fluorescent protein
ChR2: channelrhodopsin-2
PSAM: Pharmacologically selective actuator module (after [1])
PSEM^89S^: Pharmacologically selective effector molecule 89S [1]
